# Neutrophils Extracellular Traps Inhibition Improves PD-1 Blockade Immunotherapy in Colorectal Cancer

**DOI:** 10.3390/cancers13215333

**Published:** 2021-10-23

**Authors:** Hongji Zhang, Yu Wang, Amblessed Onuma, Jiayi He, Han Wang, Yujia Xia, Rhea Lal, Xiang Cheng, Gyulnara Kasumova, Zhiwei Hu, Meihong Deng, Joal D. Beane, Alex C. Kim, Hai Huang, Allan Tsung

**Affiliations:** 1 Division of Surgical Oncology, Department of Surgery, The Ohio State University, Wexner Medical Center, Columbus, OH 43210, USA; hongji.zhang@osumc.edu (H.Z.); 2007TJ0545@hust.edu.cn (Y.W.); amblessed.onuma@osumc.edu (A.O.); yujiaxia@hust.edu.cn (J.H.); hanwang@tjh.tjmu.edu.cn (H.W.); xiayaren@126.com (Y.X.); Xiang.cheng@osumc.edu (X.C.); Gyulnara.Kasumova@osumc.edu (G.K.); zhiwei.hu@osumc.edu (Z.H.); meihong.deng@osumc.edu (M.D.); Joal.Beane@osumc.edu (J.D.B.); Alex.Kim@osumc.edu (A.C.K.); 2 Institute of Pathology, Tongji Hospital, Tongji Medical College, Huazhong University of Science and Technology, Wuhan 430030, China; 3 Department of Pediatrics, Tongji Hospital, Tongji Medical College, Huazhong University of Science and Technology, Wuhan 430030, China; 4 Department of Gastroenterology, Tongji Medical College, Tongji Hospital, Huazhong University of Science and Technology, Wuhan 430030, China; 5 Neuroscience Undergraduate Division, College of Arts and Sciences, The Ohio State University, Columbus, OH 43210, USA; lal.54@buckeyemail.osu.edu; 6 Department of Microbial Infection and Immunity, Infectious Disease Institute, The Ohio State University Comprehensive Cancer Center, The Ohio State University, Columbus, OH 43210, USA

**Keywords:** neutrophils extracellular traps, programmed cell death protein 1 (PD-1), CD8+ T cells, DNase I

## Abstract

**Simple Summary:**

Despite significant advances using immune checkpoint blockade therapy for patients with advanced cancer, 40–85% of patients fail to respond to therapy. Increasing evidence suggests that the tumor immune microenvironment plays a critical role in rendering the immune checkpoint blockade ineffective. As such, targeting the tumor immune microenvironment is an attractive strategy for the treatment of solid tumors and an important approach to improve the efficacy of checkpoint inhibitors. Our study explores the combination therapy of DNase I and PD-1 antibody to prevent tumor growth. We demonstrate that this combination treatment for colorectal cancer shows superior efficacy in comparison to a single-agent treatment, both in vivo and in vitro.

**Abstract:**

Immune checkpoint inhibitors can improve the prognosis of patients with advanced malignancy; however, only a small subset of advanced colorectal cancer patients in microsatellite-instability-high or mismatch-repair-deficient colorectal cancer can benefit from immunotherapy. Unfortunately, the mechanism behind this ineffectiveness is unclear. The tumor microenvironment plays a critical role in cancer immunity, and may contribute to the inhibition of immune checkpoint inhibitors and other novel immunotherapies in patients with advanced cancer. Herein, we demonstrate that the DNase I enzyme plays a pivotal role in the degradation of NETs, significantly dampening the resistance to anti-PD-1 blockade in a mouse colorectal cancer model by attenuating tumor growth. Remarkably, DNase I decreases tumor-associated neutrophils and the formation of MC38 tumor cell-induced neutrophil extracellular trap formation in vivo. Mechanistically, the inhibition of neutrophil extracellular traps with DNase I results in the reversal of anti-PD-1 blockade resistance through increasing CD8+ T cell infiltration and cytotoxicity. These findings signify a novel approach to targeting the tumor microenvironment using DNase I alone or in combination with immune checkpoint inhibitors.

## 1. Introduction

Colorectal cancer (CRC) is the fourth most common cancer and the third leading cause of cancer-associated death worldwide [1]. The tumor microenvironment (TME) of CRC, consisting of tumor cells, immune cells, endothelial cells, and fibroblasts, plays a crucial role in tumor biology and serves as a target for novel therapeutic interventions [2,3]. Immune checkpoint inhibitors (ICI) have proven efficacy and are approved for multiple types of solid cancers, including a subset of CRC [4,5]. Programmed cell death protein 1 (PD-1) blockade induces an anti-tumor response by inhibiting the PD-1/programmed death-ligand 1 (PD-L1) checkpoint that blocks the effector functions of anti-tumor T cells [6]. Currently, pembrolizumab and nivolumab, two humanized anti-PD-1 antibodies, have shown efficacy in patients with mismatch repair (dMMR)-deficient and high microsatellite instability (MSI)-high metastatic CRC. As such, the two drugs were granted accelerated FDA approval in 2017 [5]. Unfortunately, the anti-PD-1 therapy is rendered ineffective in patients lacking immune cell infiltration [7,8,9,10]. In these patients, the survival rates remain very poor [11].

Neutrophils provide an essential front-line defense against microbes and protozoa [12,13,14] and serve as the principal cell in various conditions of inflammatory injury [15]. Neutrophils neutralize extracellular organisms by extruding their DNA via a cytolytic process, thereby trapping microbes within extracellular chromatin strands [16]. Interestingly, neutrophils play a significant role in the promotion of cancer growth in multiple tumor models [17]. It is hypothesized that the chromatin DNA, known as neutrophil extracellular trap (NET), is an important component of the tumor inflammatory infiltrate [18]. Neutrophil accumulation and infiltration are associated with tumor progression and metastases [19,20], as well as a poorer prognosis [21]. We recently reported on the role and formation of NETs in hepatocellular carcinoma (HCC) and CRC liver metastases and demonstrated that destruction of NETs using DNase I resulted in a marked reduction of HCC tumor growth and CRC liver metastases [19,20]. This established NETs as a critical factor within the tumor-promoting environment for CRC metastasis, which can be modified using DNAse I.

Currently, the effect of ICI in combination with DNase I to inhibit NETs and their positive effects on carcinogenesis are of great interest. The aim of this study was to determine the mechanisms by which the elimination of NETs using DNase I enhances the efficacy of ICI therapy in pre-clinical models of CRC. In this study, we used a murine cancer cell MC38-bearing mouse model to examine the cellular and molecular mechanisms underlying the effects of NET inhibition by DNase I in anti-PD-1 therapy. We established that inhibition of NETs by DNase I significantly improves the therapeutic effects of anti-PD-1 immune therapy in a MC38-bearing mouse model. We discovered that the combination of NET inhibition and PD-1 blockade alters the tumor immune microenvironment. Mechanistically, the inhibition of NETs with DNase I resulted in increased CD8+ T cell infiltration and cytotoxicity and ultimately overcame the resistance to anti-PD-1 monotherapy; thus, our study uncovers a novel paradigm that NET inhibition improves the efficacy of PD-1 blockade immune therapy via altering the TME.

## 2. Materials and Methods

### 2.1. Cell Culture

The murine cancer cell line of MC38 colon carcinoma (Kerafast, Boston, MA, USA) cell line was cultured in Dulbecco’s modified MEM (DMEM; Hyclone, Logan, UT, USA) with 10% fetal bovine serum (Hyclone), 2 mM glutamine (Hyclone), 0.1 mM nonessential amino acids (Hyclone), 1 mM sodium pyruvate (Hyclone), 10 mM Hepes (Thermo Fisher Scientific, Waltham, MA, USA), penicillin (100 U/mL), and streptomycin (100 μg/mL) (Thermo Fisher Scientific) at 37 °C in a 5% CO_2_ incubator (Thermo Fisher Scientific).

### 2.2. Luciferase Transfection and Bioluminescence Imaging

MC38 tumor cells expressing GFP and firefly luciferase (FLuc) genes were generated using FLuc-F2AGFP-IRES-Puro Lentivirus (Biosettia, San Diego, CA, USA) then selected with hygromycin (Thermo Fisher Scientific). For imaging, mice were anesthetized with inhaled isoflurane followed by intraperitoneal (IP) injection of potassium luciferin (300 mg/kg; Gold Biotechnology, Saint Louis, MO, USA). After 10 min to allow for luciferin distribution, mice were imaged using the IVIS Lumina II (PerkinElmer, Waltham, MA, USA) optical imaging system according to the manufacturer’s instructions. Analysis of resultant data was performed using LIVING IMAGE software (PerkinElmer). Regions of interest were manually selected and quantified for average photon flux (photons/second/cm^2^/steradian) [22].

### 2.3. Animals and Tumor Models

Wild-type (WT C57BL/6) mice were purchased from Jackson Laboratories (Bar Harbor, Maine, USA). Peptidyl arginine deiminase type IV knockout (PAD4^−/−^) mice were a gift from Dr. Yanming Wang (Pennsylvania State University, State College, PA, USA). Mice were randomly assigned to either experimental or control groups between 6 and 8 weeks of age. All mice were maintained under pathogen-free conditions. For MC38 tumor models, one million MC38 cells were subcutaneously injected on the right flank of male mice [23]. Tumor diameters were measured using calipers. The tumor volume was calculated. Anti-PD-1 or IgG2a isotype Ab (10mg/kg, Bio X Cell, Lebanon, NH, USA) and DNase I (5 mg/kg) were administered intraperitoneally starting from day 5 after tumor cell inoculation, then Anti-PD-1 or IgG2a isotype Ab was injected every 3 days and DNase I was injected daily for the duration of the experiment. Animal protocols were approved by the Animal Care and Use Committee of Ohio State University, and the experiments were performed in adherence with the National Institutes of Health Guidelines.

### 2.4. Neutrophil Isolation and In Vitro NET Formation

Murine neutrophils were isolated from bone marrow as previously described [24]. A BD Aria Plus high-speed sorter was used to sort neutrophils after incubation with PE-anti-mouse-Ly6G and APC-Cy7-anti-mouse-CD11b (BD Bioscience, Franklin Lakes, NJ, USA). To generate NETs, neutrophils were plated to adhere to coated plates for 1 h before 4 h stimulation with Calcium Ionophore (A23187, 5 µM; Sigma-Aldrich, St. Louis, MO). Neutrophils re-suspended in RPMI were also stimulated as described above for NET formation in cell culture dishes. After discarding the supernatant, NETs were harvested in 5 mL of new medium and centrifuged at 300× *g* for 10 min to pellet intact cells. Then, the supernatant was further centrifuged at 20,000× *g* for 30 min to pellet NETs. Washed NETs were then resuspended in 1 mL of RPMI 1640 with 1% BSA. Nucleosomes and cell-free DNA were measured in washed NET preparations to confirm the presence of NETs [24].

### 2.5. Quantification of NETs

To quantify NETs in mouse tumor homogenate, a capture enzyme-linked immunosorbent assay for myeloperoxidase (MPO) associated with DNA was performed as described [25,26]. For the capture antibody, a mouse MPO enzyme-linked immunosorbent assay kit (HK210-01; Hycult Biotech, Uden, Netherlands) was used according to the manufacturer’s directions. A peroxidase-labeled anti-DNA monoclonal antibody (component 2, Cell Death ELISA PLUS, Roche, Basel, Switzerland) was used. Serum nucleosome quantification was performed using the Cell Death kit [24].

### 2.6. Western Blotting

Whole-cell protein lysates from tumor were used for Western blotting. Membranes were incubated using citrullinated histone H3 (1:1000, 5103, Abcam, Cambridge, UK) and *β-actin* (1:1000, 8457S, Cell Signaling Technology, Danvers, MA, USA) as internal controls.

### 2.7. Flow Cytometry

The tumors were minced with surgical scissors and dissociated in an enzymatic solution of collagenase D (1 mg/mL) (Roche) and DNase I (100 μg/mL) for 30 min at 37 °C in a water bath with continuous agitation. After this treatment, the enzymatic solution was inactivated by adding DMEM with 10% FBS and was immediately centrifuged at 400 g for 5 min at 4 °C. The cell suspension was filtered through a 70 μm cell strainer. The cells were collected from their respective density fractions and prepared for flow cytometry analysis. One negative sample (no antibody) was used for gating purposes. Cell populations were determined by electronic gating based on forward versus side scatter. Isolated cells were incubated with fluorochrome-conjugated monoclonal antibodies, Fixable Viability Dye eFluor™ 780 (eBioscience, San Diego, CA, USA), PE-Cy-7 Rat anti-mouse CD45 (30-F11, BD Biosciences), BUV395 Rat anti-mouse Ly-6G(1A8, BD Biosciences), PerCP/Cy5.5-anti-mouse CD11b (M1/70, BioLegend, San Diego, CA, USA), APC-anti-mouse PD-1 (RMP1-30, BioLegend), Alexa Fluor 700 Rat anti-mouse CD3 (17A2, BioLegend), PerCP/Cy5.5-anti-mouse CD4 (GK1.5, BioLegend), and V450 anti-mouse CD8a (53-6.7, eBioscience). To analyze the production of IFN-γ by CD8+ T cells in the tumor, CD8+ T cells from freshly acquired tumor samples from variously treated mice were plated at a density of 2 × 10^6^ cells per well and incubated in T cell medium containing 50 ng/mL of phorbol 12-myristate 13-acetate (PMA, Sigma-Aldrich), 750 ng/mL of ionomycin, and 10 μg/mL of brefeldin (Sigma-Aldrich). Cells were incubated for 4 h at 37 °C with 5% CO_2_ followed by staining with PE anti-mouse IFN-γ Antibody (XMG1.2, BioLegend) and other cell markers. Flow cytometry data were acquired using a LSRFortessa Flow Cytometer (Becton Dickinson, Franklin Lakes, NJ, USA) with FASCDiva Software (Version 8.0.1, BD Pharmingen) and analyzed with Flowjo software (version 10). Gates and regions were placed around populations of cells with common characteristics, usually forward scatter, side scatter, and marker expression characteristics, to investigate and quantify these populations of interest [27].

### 2.8. In Vitro Cytotoxicity Assay

CD8+ T cells from the spleens of WT mice were isolated using MACS beads (Miltenyi Biotec) and stimulated for 24 h in 12-well plates (∼3 × 10^6^ cells/well) coated with anti-CD3 (4 µg/mL, Bio X Cell) and anti-CD28 (4 µg/mL, Bio X Cell) in RPMI-1640 media (Hyclone) supplemented with 10% FCS (Hyclone), penicillin (100 U/mL) and streptomycin (100 μg/mL) (Thermo Fisher Scientific), 12 mM Hepes (Thermo Fisher Scientific), and 50 µM β-mercaptoethanol (Sigma-Aldrich). Tumor cells were seeded into 96-well plates (104 cells/well) in DMEM supplemented with 10% FBS, penicillin (100 U/mL) and streptomycin (100 μg/mL), and IFN-γ (20 ng/mL, abcam) for 24 h for activation. Then, 10^4^ stimulated T cells were seeded onto activated tumor cells for 44 h before counting [28,29].

### 2.9. Quantitative Real-Time PCR

Total mRNA was extracted from CD8+ T cells with different treatments using TRIZOL reagent (Cat. 15596-026, Invitrogen, Waltham, MA, USA), and cDNA was generated from 1 μg of RNA using an RT reagent kit (Cat. k1622, Thermo Fisher Scientific). Real-time PCR was performed using the Bio-Rad CFX96 Detection System (Bio-Rad Laboratories, Hercules, CA, USA) with SYBR Green (Cat.1725121, Bio-Rad Laboratories). All reactions were run in triplicate, and relative gene expression was calculated using the comparative threshold cycle (Ct) method (relative gene expression = 2−(ΔCtsample−ΔCtcontrol)). The gene-specific primers used were forward 5′- CGGTGTCGTGTGGAACAATA -3′ and reverse 5′-TCATCATCCCAGCCGTAGTC -3′ for Prf1; forward 5′- GACCCAGCAAGTCATCCCTA -3′ and reverse: 5′- CCAGCCACATAGCACACATC -3′ for *GzmB*; forward 5′-TGATCCTTTGGACCCTCTG-3′ and reverse 5′- ACAGCCATGAGGAAGAGCTG -3′ for *Ifng*; and forward 5′-AGCCATGTACGTAGCCATCC-3′ and reverse 5′-CTCTCAGCTGTGGTGGTGAA-3′ for *β-actin*. The relative expression levels of mRNAs were normalized by the level of *β-actin* expression in each sample.

### 2.10. BioRender

Experiment outlines were created with BioRender.com (20 June 2021).

### 2.11. Statistical Analysis

The data presented in the figures are means ± SD. Survival curves were analyzed using the log-rank (Mantel–Cox) test. For experiments involving more than two groups, one-way analysis of variance with post hoc tests was used to compare the differences between treatments. The repeated measurements were assessed by ANOVA. The baseline characteristics of each group were compared using Chi-square or Fisher’s exact tests for categorical variables (GraphPad Prism version 8). All of the statistical tests used in this manuscript were two-sided and *p* < 0.05 was considered statistically significant.

## 3. Results

### 3.1. Systemic Administration of DNase Improves the Efficacy of PD-1 Blockade to Reduce the Growth of Cancer

**Figure 1:**
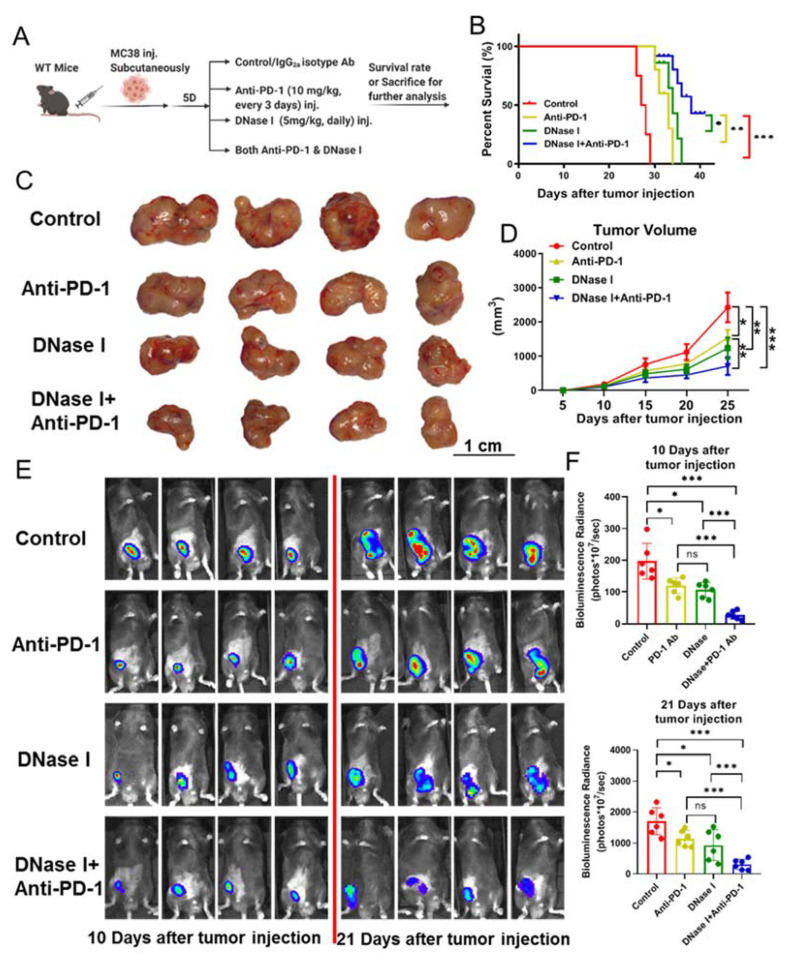
Systemic administration of DNase I improves the efficacy of PD-1 blockade to reduce the growth of cancer. (**A**) Experiment outline. C57BL/6 male mice received MC38 colorectal cancer cell injection and the mice were treated with IgG2a isotype control, anti-PD-1, DNase I or anti-PD-1, and DNase I combination. (**B**) The percentages of overall survival; *n* = 15 mice/group. (**C**) Representative photograph of flank tumor with different treatments at day 21 after tumor injection. Scale bar = 1cm; *n* = 6 mice/group. (**D**) Tumor growth curves were established by measuring flank tumors lengthwise and widthwise using the formula (d2 × D)/2 to calculate tumor volume every 2–3 days starting at day 5; *n* = 6 mice/group. ANOVA with adjustment for multiple comparisons. (**E**,**F**) The use of luciferase-labeled MC38 cells allowed in vivo tracking of tumor growth with bioluminescence imaging at day 10 and day 21; *n* = 6 mice/group. Survival curves were analyzed by log-rank (Mantel–Cox) test (**B**). The graphs show the means ± SD (**D**,**F**). ANOVA was performed with adjustment for multiple comparisons for (**D**,**F**). Note: * *p* < 0.05, ** *p* < 0.01, *** *p* < 0.001; NS: not significant. All statistical tests used in this figure were two-sided and *p* < 0.05 was considered statistically significant.

We previously demonstrated that neutrophils released NETs in the liver and lungs in response to surgical stress, nonalcoholic steatohepatitis (NASH), and tumor inoculation. Moreover, we have shown that NETs promote tumor progression and metastasis [20,22,30]. Here, we inoculated MC38 colorectal cancer cells into WT mice subcutaneously and allowed the tumor to grow for 3 weeks. The protein level of citrullinated histone H3 (Cit-H3), a marker of NET formation, was significantly increased in the tumors rather than the paired tissues from non-tumor-bearing mice (Figure S1). Inhibiting NET formation or digesting NETs with DNase I has been shown to reduce breast cancer lung metastases in mice [31]. To determine whether DNase I treatment improves the efficacy of PD-1 immunotherapy on tumor-bearing mice through elimination of NETs, we subcutaneously inoculated MC38 colorectal cancer cells into WT syngeneic mice. The mice were treated with IgG2a isotype control, anti-PD-1, DNase I or anti-PD-1, and DNase I combination (Figure 1A). Mice treated with anti-PD-1 or DNase I exhibited improved survival (Figure 1B) and reduction of tumor size, as indicated by caliper measurements, compared to the isotype-treated group (Figure 1C,D). Bioluminescent imaging consistently revealed that mice treated with anti-PD-1 or DNase I showed reductions in tumor volume compared with the control group (Figure 1E,F). Interestingly, we revealed that mice treated with the anti-PD-1 and DNase I combination showed notable improvements in survival rate (Figure 1B) and tumor volume reductions in comparison to the other three groups (Figure 1C–F). These data indicate that the administration of exogenous DNase I significantly enhances the anti-PD-1 ICIs in CRC.

### 3.2. DNase I Inhibits MC38 Tumor Cell-Induced TANS to Form NETs in Tumors

**Figure 2:**
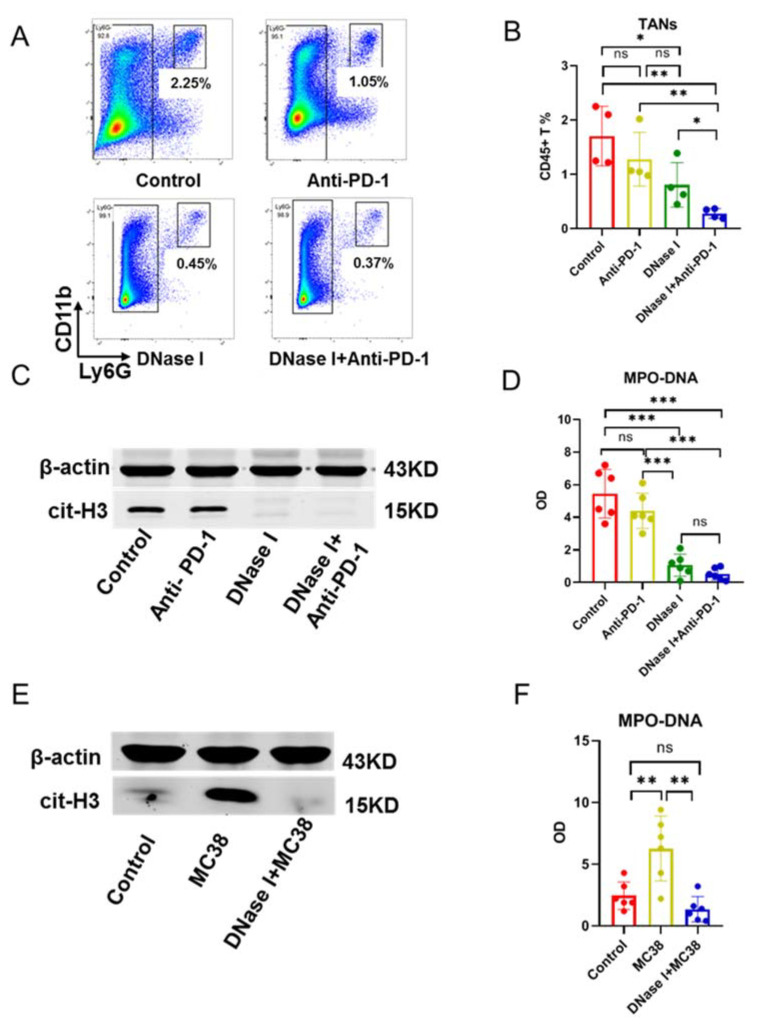
DNase I inhibits MC38 tumor cell-induced TANs to form NETs in tumors. (**A**,**B**) Percentage of TANs within the tumor from mice with different treatments; *n* = 4 mice/group from mice with different treatments; *n* = 6 mice/group. (**C**) The cit-H3 protein levels in the tumor from mice with different treatments; *n* = 6 mice/group. (**D**) MPO-DNA levels in the serum from mice with different treatments; *n* = 6 mice/group. (**E**) The cit-H3 protein levels in the neutrophils with different stimulations; *n* = 6 mice/group. (**F**) MPO-DNA levels in the medium of neutrophils with different stimulations; *n* = 6 mice/group. The graphs show the means ± SD (**B**,**D**). ANOVA was performed with adjustment for multiple comparisons for (**B**,**D**,**F**). Note: * *p* < 0.05, ** *p* < 0.01, *** *p* < 0.001; NS: not significant. All statistical tests used in this figure were two-sided and *p* < 0.05 was considered statistically significant.

Tumor-associated neutrophils (TANs) have been extensively studied in multiple steps of tumor progression and metastasis [32,33]. In addition to serving as a first line of defense in our immune system, an important role for TANs has been found in promoting tumor growth and metastasis at multiple stages of cancer progression [16,34]. To determine whether MC38 tumor cells induced TAN infiltration, we employed flow cytometry to characterize the TME in the tumors from tumor-bearing mice. Compared with control and anti-PD-1-treated groups, DNase I-treated mice exhibited significant reductions in TANs (Figure 2A,B). Notably, TANs were further decreased when treated with anti-PD-1 and DNase I combination; however, there was no difference in the infiltration of TANs in the tumor when comparing control and anti-PD-1-treated cohorts (Figure 2A,B). These data suggest that DNase I reduces MC38 tumor cell-induced TAN infiltration within tumors.

In this study, we observed a significant enhancement of anti-PD-1 treatment effect by DNase I. We hypothesized that DNase I may potentially eliminate tumor-induced NET formation to improve the PD-1 checkpoint inhibitor immunotherapies in colorectal cancer. The protein level of Cit-H3, the marker of NET formation, was remarkedly lower in the tumor tissue from the DNase I-treated and anti-PD-1 AB and DNase I combination-treated mice in comparison to control mice and those treated with anti-PD-1 AB alone (Figure 2C). Similar results were obtained when we measured the level of NETs in tumor homogenate using the MPO-DNA complex (Figure 2D). Next, we stimulated the isolated neutrophils (3 × 10^6^/well) with control (DMEM), MC38 (10^6^), or DNase I (100U/mL) [35] +MC38 for 4 h in vitro. We then measured the protein levels of Cit-H3 in neutrophils and the MPO-DNA level in the media. As expected, NET formation in neutrophils was increased with MC38 stimulation compared with the control group, while DNase I reversed the adverse effects of MC38 on neutrophils (Figure 2E). Similar results were obtained when we measured the level of NETs in media using the MPO-DNA complex (Figure 2F). Taken together, these findings suggest that DNase I inhibits tumor-induced NET formation in colorectal cancer.

### 3.3. Inhibition of NETs Improves Response to Anti-PD-1 Therapy to Anti-PD-1, Which Is Associated with Lack of CD8+ T Cell Infiltration

**Figure 3:**
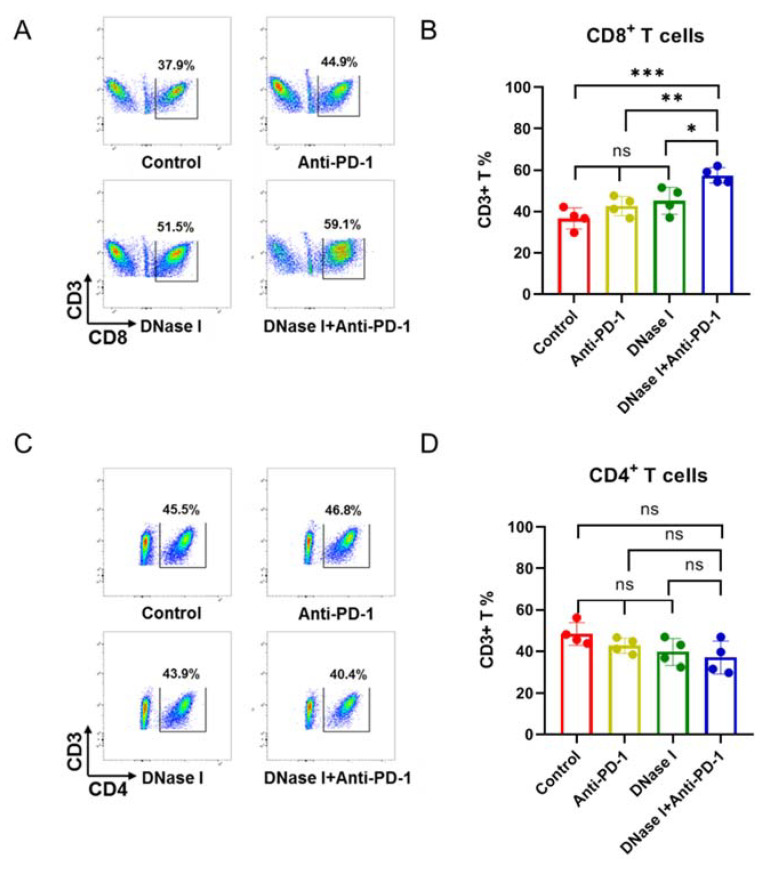
The inhibition of NETs improves the response to anti-PD-1 therapy, which is associated with a lack of CD8+ T cell infiltration. (**A**,**B**) Percentages of CD8+ T cells within the tumors from mice undergoing different treatments; *n* = 4 mice/group. (**C**,**D**) Percentages of CD4+ T cells within the tumors from mice undergoing different treatments; *n* = 4 mice/group. The graphs show the means ± SD (**B**,**D**). ANOVA was performed with adjustment for multiple comparisons for (**B**,**D**). Note: * *p* < 0.05, ** *p* < 0.01, *** *p* < 0.001; NS: not significant. All statistical tests used in this figure were two-sided and *p* < 0.05 was considered statistically significant.

We previously demonstrated that adeno-associated virus (AAV)-mediated DNase I liver gene transfer reduced the growth of colorectal liver metastases and recruited CD8+ T cells to colorectal cancer liver metastasis [22]. Adaptive immune cells in the tumor microenvironment play a crucial role in cancer cell elimination [22]. The anti-PD-1 immune checkpoint inhibitor functions by releasing the immune breaks of a pre-existing tumor-specific T cell population, such as the CD8+ T cells [6]. To identify drivers of resistance to anti-PD-1 immunotherapy during MC-38-induced colorectal cancer, we analyzed CD4+ and CD8+ T cell populations within the tumor tissue by utilizing flow cytometry. Surprisingly, the percentages of CD8+ T cells showed no significant differences among control, anti-PD-1, and DNase I-treated group (Figure 3A,B); however, the percentages of CD8+ T cells increased in the tumor following combination treatment with anti-PD-1 mAB and DNase I (Figure 3A,B). In contrast, the percentage of CD4+ T cells within the tumor treated with the combination was not significantly different from those for the other three groups (Figure 3C,D). This indicates that the inhibition of NETs improves the response to anti-PD-1 blockade, which is associated with a lack of CD8+ T cell infiltration.

### 3.4. NETs Induce the Resistance to Anti-PD-1 via Predisposition to the Cytotoxicity of CD8+ T Cells

**Figure 4:**
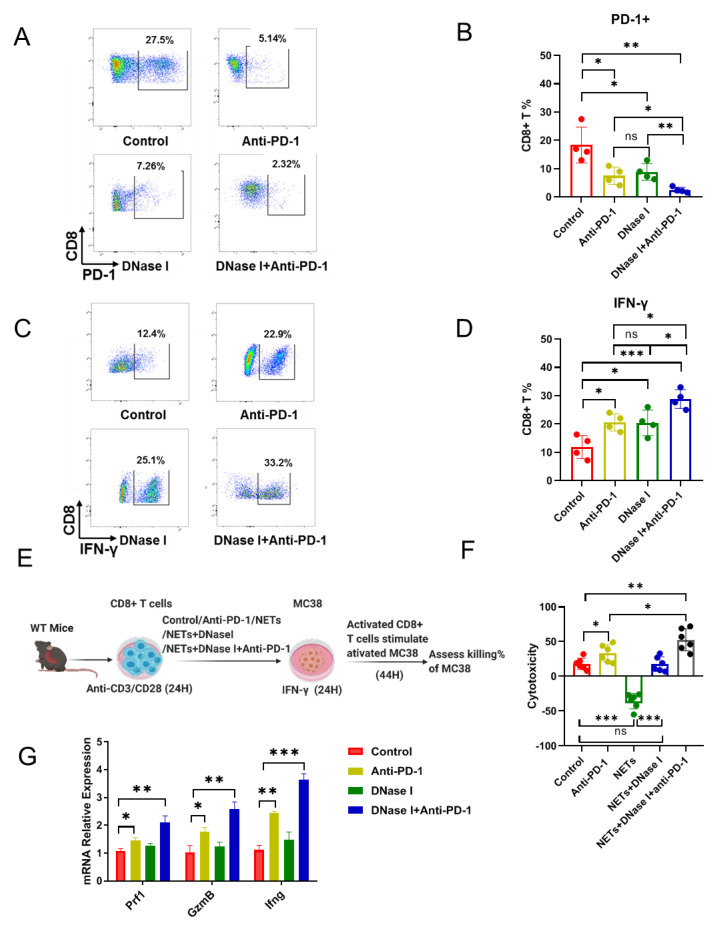
NETs induce the resistance to anti-PD-1 via predisposition to the cytotoxicity of CD8+ T cells. (**A**,**B**) Percentages of PD-1+ CD8+ T cells within the tumors from mice undergoing different treatments; *n* = 4 mice/group. (**C**,**D**) Percentages of IFN-γ+ CD8+ T cells within the tumors from mice undergoing different treatments; *n* = 4 mice/group. (**E**) Experiment outline. Anti-CD3- and anti-CD28-primed CD8+ T cells were stimulated with control or anti-PD-1 (25 μg/mL) or isolated NETs (from 2 × 10^6^ neutrophils/mL) or isolated NETs + DNase I (100U/mL) for 24 h with anti-CD3 and anti-CD28, then these stimulated CD8+ T cells were co-incubated with MC38 tumor cells. (**F**) Relative killing by CD8+ T cells of MC38 with different treatments. (**G**) Relative mRNA levels of *Prf1*, *Gzmb*, and *Ifng* in CD8+ T cells with different treatments. The graphs show the means ± SD. ANOVA was performed with adjustment for multiple comparisons for (**B**,**D**,**F**,**G**). Note: * *p* < 0.05, ** *p* < 0.01, *** *p* < 0.001; NS: not significant. All statistical tests used in this figure were two-sided and *p* < 0.05 was considered statistically significant.

Interferon (IFN)-γ acts directly on CD8+ T cells to increase their abundance. It also acts as a cytotoxic CD8+ T cell differentiation signal [36]. We hypothesized that NETs would inhibit the activation of CD8+ T cells, despite treatment with PD-1 blockade immunotherapy in CRC. We first examined the percentages of PD-1+ CD8+ T cells following treatments with either anti-PD-1, DNase I, or both. The goal was to identify a significant decrease in the expression of PD-1 and to reveal a significant decrease in antigen-specific CD8+ T cells. The combination treatment resulted in a further decrease in the percentage of PD-1+ CD8+ T cells (Figure 4A,B); however, the percentage of IFN-γ + CD8+ T cells was significantly increased when treated with either anti-PD-1 or DNase I compared with control treated group, while the combination caused a greater increase than all other treatments (Figure 4C,D). The data above show that NET formation induced by TANs may attenuate CD8+ T cell cytotoxicity. To do so, we developed an in vitro cytotoxicity assay in which we stimulated anti-CD3 and anti-CD28-primed CD8+ T cells with control or anti-PD-1 (25 μg/mL) or isolated NETs (from 2 × 10^6^ neutrophils/mL) or isolated NETs with DNase I (100 U/mL) for 24 h with anti-CD3 and anti-CD28, then co-incubated these stimulated CD8+ T cells with MC38 tumor cells (Figure 4E). CD8+ T cells mediated an increase in MC38 tumor cell death when treated with anti-PD-1 compared with control treatment. Conversely, the presence of NETs was associated with an increase in the number of MC38 tumor cells. This suggests that NETs suppress CD8+ T cell cytotoxicity, resulting in an increased number of tumor cells. Following the treatments with DNase I or DNase I plus anti-PD-1 combination, the effects of NETs on CD8+ T cells are reversed (Figure 4F). The release of IFN-γ, Prf1 (perforin), and Gzmb (granzymes) is one crucial way for CD8+ cytotoxic T cells to kill cancerous cells [37]. Next, we found that the mRNA levels of Prf1, Gzmb, and Ifn-γ were significantly increased when treated with anti-PD-1 compared with the control group, while the combination treatment caused an increase than all other treatments (Figure 4G). Together, these findings show that DNase I can improve the efficacy of anti-PD-1 by increasing the CD8+ T cell cytotoxicity.

### 3.5. Being Genetically Incapable of NET Formation Improves the Efficacy of PD-1 Blockade to Reduce the Growth of Cancer

**Figure 5:**
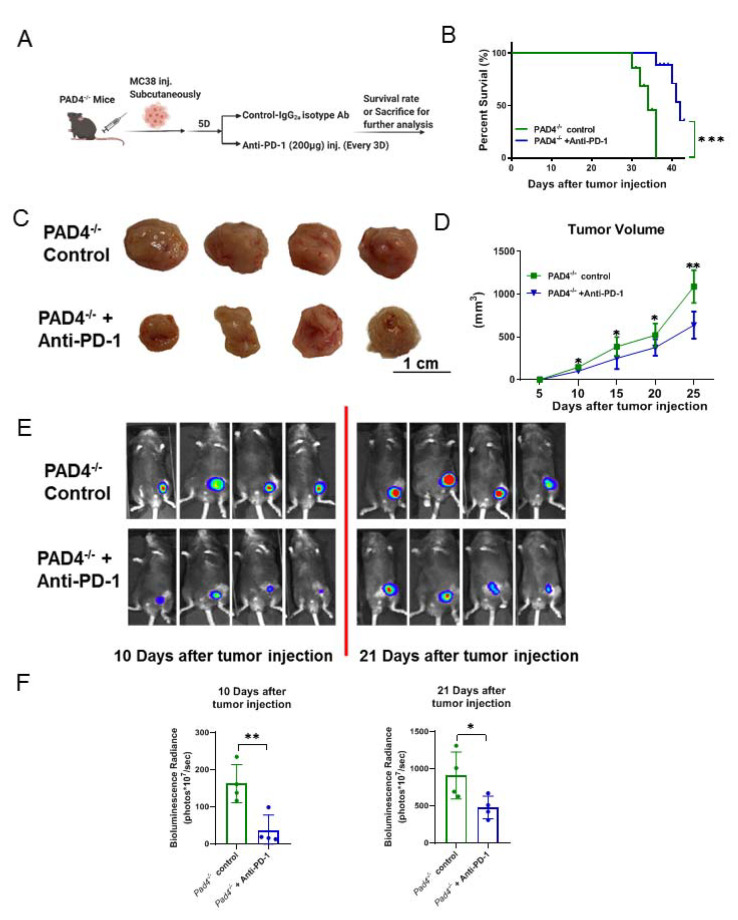
Being genetically incapable of NET formation improves the efficacy of PD-1 blockade to reduce the growth of cancer. (**A**) Experiment outline. PAD4^−/−^ male mice received MC38 colorectal cancer cell injections and the mice were treated with IgG2a isotype control or anti-PD-1. (**B**) The percentage of overall survival; *n* = 10 mice/group. (**C**) Representative photograph of flank tumor with different treatments at day 21 after tumor injection. Scale bar = 1 cm; *n* = 6 mice/group. (**D**) Tumor growth curves were established by measuring flank tumors lengthwise and widthwise using the formula (d2 × D)/2 to calculate tumor volume every 2–3 days starting at day 5; *n* = 6 mice/group. (**E**,**F**) The use of luciferase-labeled MC38 cells allowed in vivo tracking of tumor growth with bioluminescence imaging at day 10 and day 21; *n* = 6 mice/group. Survival curves were analyzed by log-rank (Mantel–Cox) test (**B**). The graphs show the ± SD. ANOVA with adjustment for multiple comparisons for (**B**,**D**). Unpaired two-sample Student’s t test for (**F**). Note: * *p* < 0.05, ** *p* < 0.01, *** *p* < 0.001; NS: not significant. All statistical tests used in this figure were two-sided and *p* < 0.05 was considered statistically significant.

Peptidyl arginine deaminase type IV (PAD4) is an enzyme that catalyzes the citrullination of histone-3, a critical step for the decondensation and expulsion of chromatin that is characteristic of NET formation [20]. PAD4^−/−^ mice are, therefore, naturally NET-deficient. Deletion of PAD4 has been shown to decrease the liver metastases that are formed from breast and colon cancers [38,39]. To further investigate this interaction, we inoculated MC38 colorectal cancer cells into PAD4^−/−^ mice subcutaneously and treated the mice with IgG2a isotype control or anti-PD-1 (Figure 5A). Mice treated with anti-PD-1 experienced improved survival (Figure 5B) and a reduction in tumor size compared to the isotype-treated group (Figure 5C,D). Bioluminescent imaging showed consistent results, whereby mice treated with anti-PD-1 showed reduced tumor size compared with the control group (Figure 5E,F). These data indicated that the abrogation of NETs with PAD4^−/−^ enhances anti-PD-1 checkpoint inhibitor immunotherapies in CRC.

## 4. Discussion

The use of ICIs has drastically changed the treatment landscape for patients with MSI-high or MMR-deficient CRCs [4,5]. Although various therapies are approved for the treatment of MSI-high or MMR-deficient CRCs, the survival rates for these patients remain very poor [11]. In this study, we utilized DNase I to inhibit NET formation in MC38 tumor-bearing mice. We demonstrated that DNase I improves the response to anti-PD-1 therapy through enhancement of CD8+ T cell infiltration and cytotoxicity.

Inflammation during tumorigenesis may impact immune surveillance or alter the TME, creating a more hostile environment for the host immune response to cancer [18,40]. Neutrophils were originally considered to act as antigen-presenting cells to promote anti-tumor T cell responses. It has been shown that sub-populations of neutrophils, called tumor-associated neutrophils, exhibit tumor-supportive functions by suppressing anti-tumor T cells [41,42,43]. TANs have been shown to be activated to release NETs, which are able to promote tumor progression and metastasis [44]. NETs also are implicated in many solid cancers, including CRC, where they are involved in cancer growth or clearance and the TME [16,45,46,47]. For example, the overproduction of interleukin (IL)-8 activates neutrophils toward NET formation and has been shown to promote CRC liver metastasis [48]. Inoculation of cancer cells in the lungs induces neutrophil infiltration and NET formation, suggesting that cancer cells themselves can induce NET formation, aiding in cancer cell adhesion and growth [38]. DNase I displays specific anti-tumor effects in several different tumor models [20,22,30,49,50]. Our studies further indicate that inhibiting NETs by DNase I administration or using PAD4^−/−^ enhances anti-PD-1 checkpoint inhibitor immunotherapies in CRC. Furthermore, DNase I reverses MC38 tumor cell-induced TAN infiltration into the tumor. We show here that there are NET formation occurs within the tumor, while MC38 tumor cells induce the neutrophils to form NETs in vitro.

IFN-γ acts directly on CD8+ T cells to increase their abundance and also acts as a cytotoxic CD8+ T cell differentiation signal. This is essential for the induction of the cytotoxic T cell precursor for proliferation during virus infection. This is critical in the process of inducing T-cell migration to tumor sites rather than generating anti-tumor protective T cells [36,51]; however, finding novel cellular and (or) molecular targets that can improve and overcome resistance to immunotherapies remains one of the main challenges that need to be resolved to increase the efficacy and outcome of cancer immunotherapies [2,52]. Anti-PD-1 therapy has been combined with various chemotherapies, small molecules, cancer vaccines, and immune-stimulatory agents [53]. Anti-PD-1 immune checkpoint inhibitor has been shown to work by unleashing the immune breaks of pre-existing tumor-specific T cell populations, especially CD8+ T cells [6]. The benefits of immunotherapeutic strategies outside of ICI have not yet been achieved in MSS or MSI-low CRCs. Further research is urgently needed to clarify a mechanism that can be further built upon in the MSI-high subset of CRC. Instead of single therapy, the combination of standard cancer therapies has been shown to improve the response rate [54,55,56,57]. Our previous work has shown that AAV-mediated DNase I liver gene transfer reduces the growth of CRC liver metastases and recruits CD8+ T cells to CRC liver metastasis [22]; therefore, developing combination therapies, such as PD-1 blockade with DNase I treatment, might lead to treatment of cancer patients who receive the least benefit from monotherapy.

Here, we show that DNase I improves the efficacy of anti-PD-1 by increasing CD8+ T cell infiltration. Moreover, our results indicate that the combination of DNase I and anti-PD-1 increases CD8+ T cell cytotoxicity. We also use PAD4^−/−^ mice, which are genetically incapable of NET formation, to validate the in vivo experiment. Similar to the DNase I treatment, removing NETs from the TME using PAD4^−/−^ mice improved the efficacy of anti-PD-1 ICI therapy, similarly to the DNase I combination therapy, resulting in a decrease in tumor growth and an improvement in overall survival rate; thus, our study provides mechanistic insights into combination therapy and uncovers the improvement in CD8+ T cell infiltration and cytotoxicity mediated by DNase I during ICI therapy. While these results are important, further studies are warranted to investigate whether and how NET inhibition is involved in the flux of CD8+ T cell infiltration and to investigate the improved cytotoxic function observed here. One possibility may be through the use of increased DNA sensing by TLR9 on CD8+ T cells in mice treated with DNAse I. It has been demonstrated that the TLR9 agonist effectively increases tumor infiltration by CD8+T cells [58]. Furthermore, the cGAS-STING pathway could sense the extracellular DNA and become activated. It has been reported that activation of STING signaling activates immune-suppressive cells [59,60]. In addition, DNase I may cleave extracellular DNA, thereby inhibiting the cGAS-STING pathway and ultimately inhibiting immune-suppressive cells such as regulatory T cells. Furthermore, tumor-accumulating neutrophils can produce NETs enriched with PD-L1, thereby inhibiting cytokine production and the proliferative capacity of tumor-infiltrating lymphocytes through the PDL-1/PD-1 axis. DNase I treatment reverses the functionality of tumor-infiltrating lymphocytes.

In summary, our data suggest a potential new therapeutic strategy to overcome PD-1 blockade resistance. We found that TANs and NET formation were enriched in the tumor tissue in a MC38-bearing mouse model, constituting a target to reverse PD-1 blockade resistance. Moreover, inhibition of NETs improves the response to anti-PD-1 therapy, which is associated with a lack of CD8+ T cell infiltration and the suppression of CD8+ T cell cytotoxicity. These findings open potential avenues for the targeting of the tumor immune microenvironment, either alone or in combination with immune checkpoint inhibitors, which might benefit patients with advanced cancer in the future.

## Data Availability

The data presented in this study are available in this article and Supplementary Figure S1. All other data that support the findings of this study are available from the corresponding author upon request.

## Supplementary Materials

The following are available online at https://www.mdpi.com/article/10.3390/cancers13215333/s1: Figure S1: NETs are involved during tumor progression.
