# Supplementary material for: Neutrophils Extracellular Traps Inhibition Improves PD-1 Blockade Immunotherapy in Colorectal Cancer
Source: Cancers (Basel). 2021 Oct 23;13(21):5333. doi: 10.3390/cancers13215333 (PMC8582562; doi:10.3390/cancers13215333)
Supplement: Supplementary file 1 [file cancers-13-05333-s001.zip › cancers-1406203-supplementary.pdf]

cancers

Supplementary Materials: Neutrophils Extracellular Traps Inhibition Improves PD-1 Blockade Immunotherapy in Colorectal Cancer

Hongji Zhang, Yu Wang, Amblessed Onuma, Jiayi He, Han Wang, Yujia Xia, Rhea Lal, Xiang Cheng, Gyulnara Kasumova, Zhiwei Hu, Meihong Deng, Joal Beane, Alex C. Kim, Hai Huang and Allan Tsung

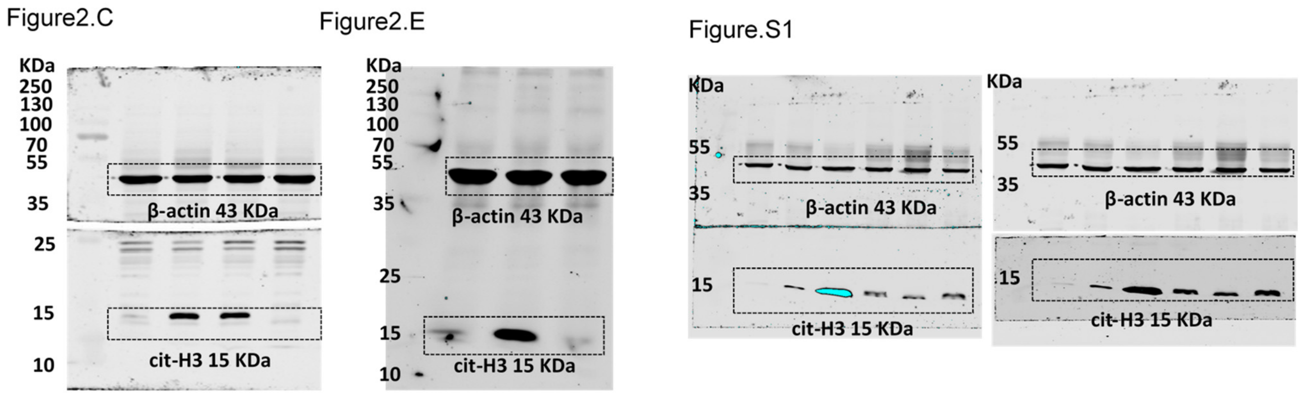

Figure S1. NETs are involved during tumor progression. The cit-H3 protein levels in the normal tumor tissue samples from mice who received MC38 injections.
